# Abscisic and Jasmonic Acids Contribute to Soybean Tolerance to the Soybean Aphid (*Aphis glycines* Matsumura)

**DOI:** 10.1038/s41598-018-33477-w

**Published:** 2018-10-11

**Authors:** Kaitlin M. Chapman, Lia Marchi-Werle, Thomas E. Hunt, Tiffany M. Heng-Moss, Joe Louis

**Affiliations:** 1 Department of Entomology, University of Nebraska-Lincoln, Lincoln, NE 68583 USA; 2 Department of Biochemistry, University of Nebraska-Lincoln, Lincoln, NE 68583 USA

## Abstract

Plant resistance can provide effective, economical, and sustainable pest control. Tolerance to the soybean aphid has been identified and confirmed in the soybean KS4202. Although its resistance mechanisms are not fully understood, evidence suggests that enhanced detoxification of reactive oxygen species (ROS) is an active system under high aphid infestation. We further explored tolerance by evaluating the differences in constitutive and aphid-induced defenses in KS4202 through the expression of selected defense-related transcripts and the levels of the phytohormones abscisic acid (ABA), jasmonic acid (JA), JA-isoleucine (JA-Ile), cis-(+)-12-oxo-phytodienoic acid (OPDA), and salicylic acid (SA) over several time points. Higher constitutive levels of ABA and JA, and basal expression of ABA- and JA-related transcripts were found in the tolerant genotype. Conversely, aphid-induced defenses in KS4202 were expressed as an upregulation of peroxidases under prolonged aphid infestation (>7 days). Our results point at the importance of phytohormones in constitutive defense in KS4202 tolerance to the soybean aphid. Understanding the underlying mechanisms of tolerance will assist breeding for soybean with these traits, and perhaps help extend the durability of *Rag (Resistance to Aphis glycines)*-mediated resistance genes.

## Introduction

Insects are important pests of plants that cause substantial loss in plant productivity and fitness. In response to insect feeding, plants have evolved a wide array of defense strategies to protect themselves against herbivore attack. Plant resistance to insects can be categorized into three main categories: antibiosis, antixenosis, and tolerance^1^. Plants exhibiting antibiosis negatively affect an insect’s biology upon feeding, resulting in increased mortality, reduced longevity, and reduced fecundity. Antixenosis, or non-preference, affects the insect’s behavior to deter or reduce colonization on resistant plants. Tolerance, a less understood plant resistance category, is composed of multiple plant characteristics. Relative to susceptible hosts, tolerant plants can maintain insect populations while withstanding or recovering from herbivore injury and still yielding significantly more biomass^1^. In contrast to the other plant resistance categories, which have a direct effect on the insect’s biology or behavior, tolerance does not impose the same selection pressure on the insect, therefore the likelihood of biotype emergence is generally considered minimal^1,2^.

Hemipterans, within the family Aphididae, are one of the most economically important insect pests on plants worldwide, representing 26% of the 45 major crop pests of main food crops in temperate climates^3^. Aphids can build extraordinary populations due to high reproductive potential (parthenogenesis), short generation times, and the high proportion of viviparous females in the population. Additionally, short-term changes in population size, as well as deprivation of host nutrition, induce the formation of winged forms that disseminate by wind currents over long distances^4^. Because the interaction of demographic factors impose high variability in aphid build up, aphid management is particularly challenging. For several years, farmers have been using insecticides as one of the major tactics to control aphids. However, the continued reliance on insecticides has led to the loss of aphid resistance in field-grown crops, and leading to cascading negative effects on non-target and beneficial organisms and environmental safety^5–8^. Alternatively, aphid management can be best performed by blending plant resistance with integrated pest management (IPM)^9–11^.

Arthropod-tolerant plants blend well with IPM, as it exerts minimal negative impacts on the targeted pest as well as most natural enemies, and may have higher economic injury levels (EILs) relative to susceptible genotypes^12,13^. Higher EILs generally translate into a delayed chemical control, reducing the need for frequent chemical applications^14^. Despite the evident benefits of implementing tolerance for mitigating insect damage, the absence of detailed knowledge in the mechanisms and genetics underlying plant tolerance limits its incorporation in breeding programs and IPM.

Existing research on tolerance to hemipteran pests emphasize the upregulation of photosynthetic activity and reactive oxygen species (ROS)-detoxification mechanisms^15^. In plants, redox control is governed by enzymes and antioxidants that rapidly detoxify ROS. Environmental stressors, such as insect feeding, may cause oxidative bursts leading to ROS accumulation, and ultimately cell toxicity and death. Several studies have indicated that insect feeding increases localized and plant-wide peroxidase activity, and that this activity is higher in tolerant plants^16–19^.

The current literature on compatible and incompatible interactions between plants and several aphid species has uncovered that plants defend themselves using a variety of defense signaling pathways. Many of these interactions are dependent on several hormonal pathways, including but not limited to salicylic acid (SA), jasmonic acid (JA) and ethylene (ET) signaling^20–22^. Plant receptors recognize aphid-feeding via elicitors, which may be derived from an aphid’s saliva or even products of endosymbiotic bacteria^23–25^. Upon aphid probing, a cascade of defense reactions occurs via the recognition of aphid elicitors, where calcium- and ROS- related signaling play important roles in triggering the activation of defense pathways such as JA, SA, and ET^21,26^.

Accumulation of SA as a response to aphid colonization has been documented in multiple plant systems and is credited with being important in plant resistance to phloem-feeding insects^27–29^. SA also mediates localized plant tissue hypersensitive and systemic acquired responses, and induces the expression of defense responsive transcripts, including pathogenesis-related (*PR*) genes and proteins. In soybean, *Glycine max* (L.) Merrill, *PR1* was highly expressed in aphid resistant *Rag1* (***R****esistance to* ***A****phis* ***g****lycines1*) plants infested with soybean aphids (*Aphis glycines* Matsumura), when no changes occurred in the susceptible genotype^30,31^. Studies have also shown that phloem feeding insects may induce JA-associated transcripts as well, although this pathway is better characterized in plants stressed by chewing insects^32,33^. Aphid-infested wheat (*Triticum aestivum*) and barley (*Hordeum vulgare*) have also induced JA-associated transcripts including lipoxygenases (*LOX*), coronatine-insensitive1 (*COI1*), 12-oxophytodienoate reductase (*OPR*) and cytochrome P450^34,35^. In addition, abscisic acid (ABA) plays an important role in abiotic stress tolerance and pathogen resistance though its role in insect-plant interactions is less understood.

The soybean aphid (*Aphis glycines* Matsumura) is considered as the most economically important pest of soybean in the U.S.^3^. Upon introduction of the soybean aphid from Asia in 2000, the application of chemical insecticides, development of economic thresholds and injury levels, and deployment of aphid-resistant cultivars have been the primary methods for controlling soybean aphids^5,6,36^. Traditional aphid-resistant soybeans contain one or more dominant *Rag* genes, which exhibit antibiosis and/or antixenosis, that deters aphid feeding and subsequent damage^37–40^. However, the sustainable implementation of these traits faces challenges due to the emergence of soybean aphid biotypes^41–43^.

Soybean tolerance to the soybean aphid was observed in the soybean genotype KS4202^44–46^. Tolerance in KS4202 was demonstrated in other studies where aphid feeding reduced soybean yield at a rate of 3.1% for every 10,000 aphid-days that accumulates^14,45,46^. Conversely, the same comparison in susceptible soybean resulted in yield losses of ~7%^36^. Defensive mechanisms in KS4202 may be composed of metabolic changes that involve oxidative enzymes and a primed photosynthetic system^16,17^. Functional transcriptomic approaches revealed a wide variety of responses induced by soybean aphids in KS4202, including the overexpression of peroxidases, cytochrome P450s, and WRKY transcription factors in the tolerant soybean^47^. Two peroxidase transcripts, *Peroxidase 52* (*PRX52*) and *Ascorbate peroxidase 4* (*APX4*), were reported to be significantly induced by soybean aphids in KS4202 at 15 days post-infestation (dpi). KS4202 also exhibits tolerance to *Bemisia tabaci* biotype B via unknown factors that are independent of the oxidative enzymes, such as superoxide dismutase, polyphenoloxidase, and peroxidases^48^.

While the upregulation of peroxidase activity in aphid-tolerant soybean is generally understood, other defense mechanisms remain unknown. Additional knowledge may help to identify phenotypic characteristics linked to tolerance that will assist breeding of tolerant plants. In this study, we evaluated the differences in constitutive and induced responses between tolerant KS4202 and susceptible soybeans by monitoring the expression of selected defense-related transcripts, including peroxidases, phytohormone-associated transcripts and quantifying the levels of ABA, JA, and SA over the time of aphid infestation. Finally, we propose a working hypothesis for soybean aphid tolerance in KS4202.

## Results

### Aphid colonization pattern in KS4202 is similar to aphid-susceptible soybeans

**Figure 1:**
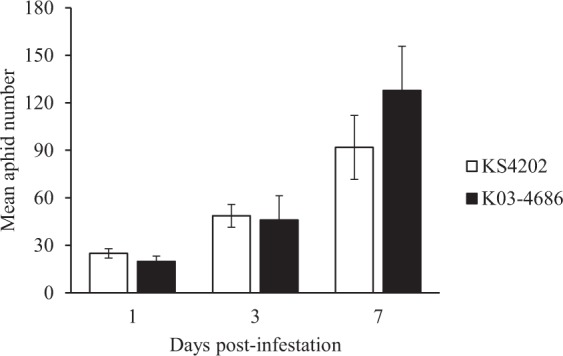
KS4202 is susceptible to aphid colonization. Mean aphid number (±SE) for infested soybean in the late response study (N = 5).

To demonstrate the similarities in aphid colonization between KS4202 and susceptible soybean, total soybean aphid numbers (adults and nymphs) were recorded in the late response study (Fig. 1). Consistent with previous studies^17,47^, aphid population numbers were similar between soybean genotypes and the brief duration of soybean aphid infestation did not result in visual damage (i.e. visual damage = 1) for either tolerant or susceptible soybean.

### KS4202 exhibits higher constitutive levels of ABA and JA

**Figure 2:**
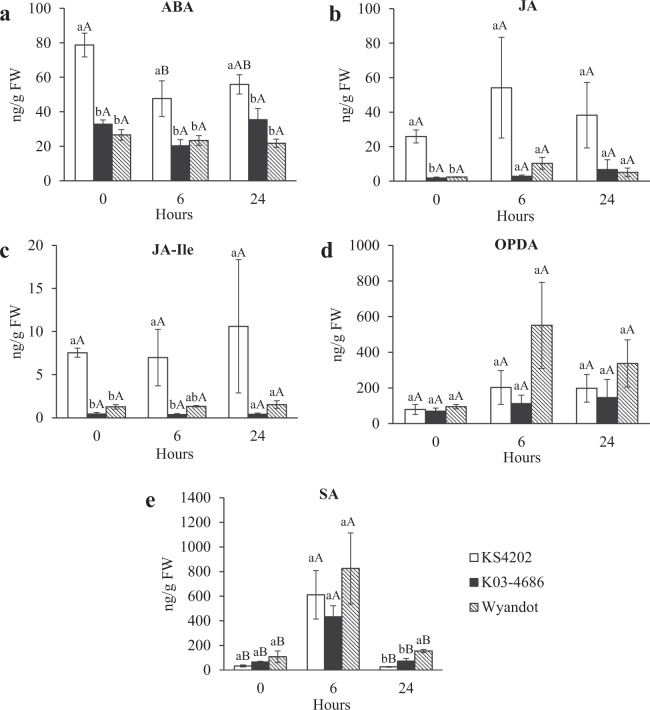
KS4202 shows higher constitutive levels (ng/g fresh weight [FW]) of ABA, JA, and JA-Ile. ABA, SA, JA, JA-Ile, and OPDA levels in tolerant KS4202 and susceptible K03-4686 and Wyandot soybean (N = 3). Different lowercase letters indicate significant differences between genotypes within the same treatment (*P* < 0.05). Different uppercase letters indicate significant differences between treatments within the same genotype (*P* < 0.05). Error bars represent mean ± SE.

To assess both constitutive and aphid-induced differences in phytohormone levels between KS4202 and susceptible soybeans, a time-course experiment was performed in which plant tissue (V3 stage) was harvested pre- and at 6 and 24 hours post-infestation (hpi). Tolerance in KS4202 is well defined during this stage and continues to be expressed as soybean plants enter reproductive stages^39^. Our results indicate that KS4202 had higher levels of ABA within each time point analyzed compared to aphid-susceptible soybeans (Fig. 2a). The constitutive levels of ABA in the tolerant soybean were over 2x greater than K03-4686 (*P* = 0.0004) and Wyandot (*P* = 0.0002) while similar in both susceptible soybeans (*P* = 0.3693). After aphid infestation, ABA levels decreased in KS4202 at 6 hpi (*P* = 0.0313) but remained higher compared to both susceptible genotypes K03-4686 (*P* = 0.0245) and Wyandot (Fig. 2a; *P* = 0.0382). There were no significant differences in the ABA levels before and after infestation in both aphid susceptible soybeans .

Similarly, constitutive levels of both JA and JA-Ile were higher in the tolerant soybean (Fig. 2b,c; JA: *P* = 0.0003, JA-Ile: *P* < 0.0001). While not statistically significant, levels of JA in KS4202 were also higher than susceptible genotypes at 6 (K03-4686: *P* = 0.0759; Wyandot: *P* = 0.1168) and 24 hpi (K03-4686: *P* = 0.102; Wyandot: *P* = 0.0886) with similar trends seen with JA-Ile. Aphid feeding induced no noticeable changes in the accumulation of these hormones in any genotype (Fig. 2b,c; *P* > 0.05). Endogenous levels of the JA precursor, cis-(+)-12-oxo-phytodienoic acid (OPDA), were similar across all soybeans tested (Fig. 2d; *P* > 0.05). Furthermore, our results indicate that constitutive levels of SA do not appear to be different between tolerant and susceptible genotypes (Fig. 2e; *P* = 0.21); however, aphid infestation resulted in higher SA levels at 6 hpi in all soybean genotypes (Fig. 2e; KS4202: *P* = 0.0168; K03-4686: *P* = 0.0041; Wyandot: *P* = 0.0421).

**Figure 3:**
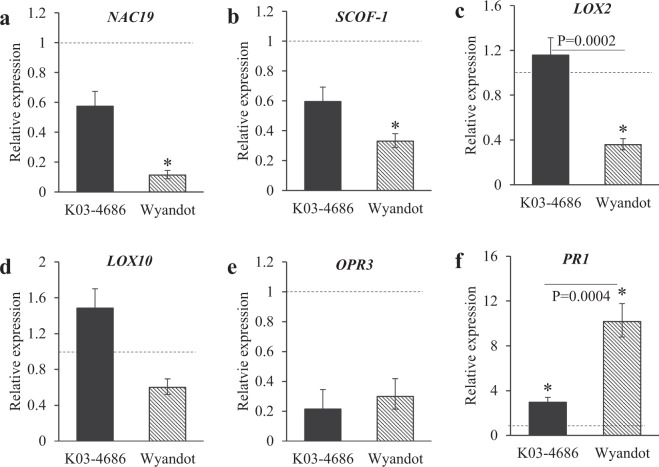
Constitutive (i.e., aphid uninfested plants) expression of JA-, SA-, and ABA-related transcripts in aphid-susceptible soybeans relative to aphid-tolerant KS4202 (N = 5). Baseline expression in KS4202 is 1 for each genotype. (*) indicates a significant difference (*P* < 0.05) between susceptible and KS4202. Error bars represent mean ± SE.

To further evaluate constitutive differences related to tolerance, additional studies focused on the relative expression of JA-, SA-, and ABA-related transcripts. The same biological replicates used for hormone analysis were used to test constitutive and aphid-induced expression at early feeding time points (6 and 24 hpi). A second study evaluated the expression of the same transcripts at later aphid infestation time points (1, 3, and 7 dpi). The NAC domain protein *NAC19* (previously described as *ARABIDOPSIS TRANSCRIPTION ACTIVATING FACTORS1* or *ATAF1*) and soybean cold‐inducible zinc finger transcription factor, *SCOF-1*, were used as ABA-responsive marker transcripts^49,50^. Overall, constitutive expression of both *NAC19* and *SCOF-1* were higher in KS4202 relative to aphid-susceptible soybeans (Fig. 3a,b; *P* < 0.0001).

There were also significant interactions between soybean genotype × aphid treatment (*P* = 0.0028) and genotype × hpi (*P* < 0.0001) treatments for *NAC19*. In KS4202, a sharp increase in expression of this transcript occurred at 6 hpi (*P* < 0.001) followed by a decrease at 24 hpi (*P* = 0.02) in infested plants relative to uninfested. Aphid-mediated suppression of *NAC19* was also seen at both early time points in the susceptible, K03-4686, although no notable changes occurred with Wyandot (Table 1). Expression of *SCOF-1* did not change in tolerant plants as a response to aphid feeding and was suppressed at 24 hpi in K03-4686 (*P* = 0.001) and 6 hpi in Wyandot (*P* = 0.08). Expression of both *NAC19* and *SCOF-1* were not evaluated at 1 and 3 dpi in the late-response study; however at late infestation (7 dpi) aphid feeding triggered 2.53x relative fold change of *NAC19* (*P* = 0.05) in KS4202.

**Table 1:** Early response study: Aphid-induced expression of JA-, SA- and ABA-related transcripts at 6 and 24 hpi (N = 5).

		KS4202 (tolerant)		K03-4686 (susceptible)		Wyandot (susceptible)	
		6 hpi	24 hpi	6 hpi	24 hpi	6 hpi	24 hpi
JA	*LOX2*	0.42 ± 0.06*	0.79 ± 0.10	1.09 ± 0.13	1.13 ± 0.15	0.59 ± 0.13	0.62 ± 0.11
	*LOX10*	2.13 ± 0.28	1.46 ± 0.15	3.87 ± 0.68	1.74 ± 0.23	0.15 ± 0.04*	1.43 ± 0.28
SA	*PR1*	0.46 ± 0.07*	1.39 ± 0.16	1.02 ± 0.12	0.95 ± 0.11	0.94 ± 0.17	1.37 ± 0.23
ABA	*NAC19*	28.55 ± 3.40*	0.05 ± 0.01*	0.23 ± 0.02*	0.10 ± 0.01*	1.21 ± 0.10	0.79 ± 0.10
	*SCOF-1*	1.61 ± 0.14	0.75 ± 0.14	1.69 ± 0.25	0.17 ± 0.02*	0.40 ± 0.03	1.55 ± 0.21

Baseline expression in uninfested soybean is 1 for each genotype. (*) indicates a significant difference (*P* < 0.05) between the infested and uninfested control soybean. Error bars represent mean ± SE.

Two lipoxygenases, *LOX10* previously found to be differentially expressed in KS4202^47^ and *LOX2*, a chloroplastic-like linoleate 13S-lipoxygenase 2 in the JA-biosynthesis pathway of soybean, and *OPDA-REDUCTASE 3* (*OPR3*) were used as markers for JA-related transcript expression. The constitutive expression of all three transcripts showed varying changes in both early and late response studies. The expression of *LOX2* was higher in KS4202 relative to the susceptible Wyandot (Fig. 3c; *P* = 0.0006). Similarly, constitutive expression of *LOX10* in KS4202 was higher than the susceptible soybeans, although not statistically significant (Fig. 3d; *P* = 0.40). While both transcripts were upregulated in compared to Wyandot, no notable differences occurred between KS4202 and K03-4686 (Fig. 3c, *LOX2*: *P* = 0.61; Fig. 3d, *LOX10*: *P* = 0.65). Additionally, no trends in aphid-induced changes were seen for these transcripts at early and late infestation time points (Tables 1 and 2) other than an initial suppression of *LOX2* in KS4202 (*P* = 0.003) and *LOX10* in Wyandot at 6 hpi (*P* = 0.03; Table 1). In KS4202, constitutive expression of *OPR3* was higher relative to both susceptible soybeans (Fig. 3e; K03-486: *P* = 0.07; Wyandot: *P* = 0.14). Aphid-induced changes in *OPR3* expression were not evaluated in the early response study, however, the late response study indicated that this transcript was only induced in KS4202 at 7 dpi (Table 2; *P* = 0.047).

**Table 2:** Late response study: Aphid-induced expression of JA-, SA- and ABA-related transcripts at 1, 3 and 7 dpi (N = 5).

		KS4202 (tolerant)			K03-4686 (susceptible)		
		1 dpi	3 dpi	7 dpi	1 dpi	3 dpi	7 dpi
JA	*LOX2*	0.80 ± 0.05	1.13 ± 0.12	1.32 ± 0.10	1.52 ± 0.27	2.71 ± 0.29	2.40 ± 0.13
	*LOX10*	0.43 ± 0.08	1.11 ± 0.39	1.18 ± 0.10	1.78 ± 0.21	1.77 ± 0.31	1.98 ± 0.29
	*OPR3*	1.67 ± 0.16	0.59 ± 0.09	2.21 ± 0.15*	1.06 ± 0.08	0.58 ± 0.04	1.92 ± 0.16
SA	*PR1*	1.13 ± 0.05	0.60 ± 0.02	6.02 ± 0.73*	2.96 ± 0.15	0.23 ± 0.02	4.42 ± 0.21
ABA	*NAC19*	—	—	2.53 ± 0.19*	—	—	0.78 ± 0.10
	*SCOF-1*	—	—	0.43 ± 0.04	—	—	1.38 ± 0.21

Baseline expression in uninfested soybean is 1 for each genotype. (*) indicates a significant difference (*P* < 0.05) between the infested and uninfested control soybean. Error bars represent mean ± SE.

### Soybean aphid feeding triggers SA in soybeans

Although no constitutive differences of SA between tolerant and susceptible soybeans were observed (Fig. 2e; *P* = 0.21), all soybeans responded to aphid feeding with higher SA levels at 6 hpi (*P* < 0.0001). Interestingly, in our study, constitutive expression of *PR1* was higher in both susceptible soybeans relative to KS4202 (Fig. 3f; K03-4686: *P* = 0.0013; Wyandot: *P* < 0.0001). Upon aphid introduction, *PR1* was suppressed even more in KS4202 at 6 hpi (Table 1; *P* = 0.02), before returning to uninfested expression levels at 24 hpi (Table 1; *P* = 0.34). Aphids elicited no notable early-response changes in Wyandot (Table 1). In the late response study, expression of *PR1* behaved similarly in the susceptible K03-4686 and tolerant KS4202, with aphid-feeding slightly down-regulating expression at 3 dpi (Table 2; *P* = 0.41) and increasing expression at 7 dpi (Table 2; *P* = 0.008).

### Soybean tolerance to aphids is independent of *PHYTOALEXIN DEFICIENT4*

Previously, it was shown that *Glycine max PHYTOALEXIN DEFICIENT4* (*GmPAD4*) is required for providing resistance to pathogens and aphids^51,52^. Our results indicate that the expression of *GmPAD4* was constitutively higher in the susceptible soybeans (Supplemental Fig. 1a; *P* = 0.0005). Conversely, in KS4202, aphids induced a 2x fold-change in *GmPAD4* expression at 6 hpi (*P* = 0.0008) before returning to uninfested levels at 24 hpi (Supplemental Fig. 1b). Suppression of *GmPAD4* in infested K03-4686 occurred at 24 hpi (*P* = 0.04), although not significant at 1 dpi in the late response study (*P* = 0.24). At 3 dpi, aphids also induced *GmPAD4* in K03-4686 (Supplemental Fig. 1c).

### Peroxidase activity is upregulated in KS4202 after several days of aphid infestation

**Figure 4:**
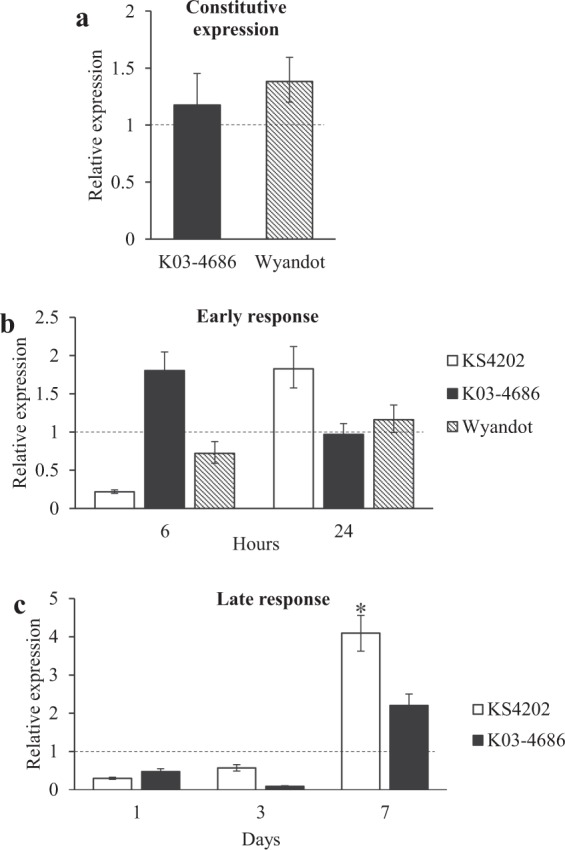
Constitutive and aphid-induced expression of *PRX52*. (**a**) Constitutive expression of *PRX52* in aphid-susceptible soybean genotypes relative to aphid-tolerant KS4202 with a baseline expression of 1. (**b**) Early response study – Relative expression of *PRX52* in aphid-infested plants at 6 and 24 hpi (**c**) Late response study – Relative expression of *PRX52* in aphid-infested plants at 1, 3, and 7 dpi. Baseline expression in uninfested soybean is 1 for each genotype. Five biological replicates per treatment combination were used. (*) indicates a significant difference between treatments and control. Error bars represent mean ± SE.

Using next generation sequencing, the transcript *Peroxidase 52* (*PRX52*), had a 2.6 log_2_ fold change in KS4202 at 15 dpi^47^. While no differences in the constitutive expression of *PRX52* among the soybeans were observed (Fig. 4a), aphid infestation suppressed *PRX52* in KS4202 at 6 hpi (Fig. 4b, *P* = 0.003). The results for early and late response at 24 hpi, and 1 and 3 dpi, respectively, indicate no differences in the expression of this transcript upon aphid introduction in either soybean (Fig. 4b,c; *P* > 0.05). However, at 7 dpi, *PRX52* was significantly upregulated in infested KS4202 when no changes were observed in the susceptible soybean (Fig. 4c; *P* = 0.049). To expand on these findings, an enzyme assay for peroxidase activity within plant tissues before and after aphid infestation at earlier time points (i.e., 6 and 24 hpi) was performed. Similar to the aforementioned results, no differences in constitutive activity or aphid-induced changes were observed (*P* > 0.05; Table 3).

**Table 3:** Total peroxidase activity (μmol/min/mg protein) for soybeans at pre-aphid infestation (0 h) and post-aphid infestation (6 and 24 hpi).

Genotype	0 h	6 hpi		24 hpi	
	Control	Control	Infested	Control	Infested
KS4202	20.02 ± 2.90	24.50 ± 5.75	24.16 ± 4.61	22.69 ± 5.01	24.93 ± 8.32
K03-4686	24.11 ± 7.41	21.50 ± 4.40	23.48 ± 6.22	31.12 ± 7.98	24.44 ± 4.16
Wyandot	29.00 ± 11.55	21.57 ± 6.82	22.88 ± 5.99	23.13 ± 4.17	22.25 ± 11.87

Five biological replicates per genotype were used. (*) indicates a significant difference between treatments. Error bars represent mean ± SE.

## Discussion

To date, multiple studies have investigated changes in JA, SA, and ABA in susceptible and *Rag*-mediated resistant soybean challenged by soybean aphids^30,31,53,54^, however, none documented these parameters in aphid tolerant soybean. Because host resistance to insects is a relative measure^55^ and KS4202 is so far, the sole genotype categorized as tolerant to the soybean aphid, the phytohormone levels and related gene expression were relative to two aphid-susceptible soybeans (K03-4686 and Wyandot). Combined with former studies on the role of induced response of peroxidases^16,17^, here we highlight the importance of constitutive levels of ABA, JA and JA-Ile phytohormones as mechanism for aphid-tolerance in soybean.

While the role of ABA in plant abiotic stress responses and pathogen resistance is well documented^56,57^, its specific role in plant-insect interactions is less understood. Studham and MacIntosh (2013) previously reported that soybean aphid infestation induces ABA responses in susceptible soybean^31^. Further studies of this susceptible system have supported the hypothesis that soybean aphids induce ABA-related transcript expression when SA-mediated defenses accumulate^54^. Here, we report higher constitutive levels of endogenous ABA and expression of the ABA-related transcripts *NAC19* and *SCOF-1* in aphid-tolerant soybean. Based on these evidences, we hypothesize that the upregulation of ABA in KS4202 is required for the genotype’s susceptibility to aphid colonization, and that other evaluated factors such as JA and peroxidase activity contribute to tolerance of aphid-induced damage.

Plant perception of herbivores generates diverse defense responses that include interactions among several phytohormone pathways. Early models have suggested the important role of ABA as a synergist required for the induction of JA in response to herbivore wounding^58^; however, the exact relationship between ABA and JA appears to be dependent upon the specific plant-insect system. Thaler and Bostock (2004) reported no changes in JA expression between wild-type and ABA-deficient tomato plants, though ABA appeared to be a key player in reducing *Spodoptera exigua* caterpillar growth^56^. While high constitutive levels of JA and JA-Ile in KS4202 mirrored ABA, up-regulation of JA-related transcripts in response to aphid feeding were observed in susceptible soybean plants. The greater induction of these transcripts in the susceptible genotypes appears to be related to their overall lower constitutive expression relative to KS4202, which displayed minimal aphid-induced changes in *LOX* expression. Previous research has shown that the induction of *LOX* transcripts in response to aphids occurs rapidly (12–36 hours) in plants that exhibit anitbiosis^59^. Additionally, Marimuthu and Smith (2012) observed that JA-related genes were more highly induced in susceptible barley than in barley tolerant to Russian wheat aphid (*Diuraphis noxia*), when majority of the same transcripts were constitutively higher in tolerant plants^60^. Phytohormone analysis of susceptible and *Rag*-mediated resistant soybean also reported an induction of JA in susceptible genotypes after 1 and 7 days of soybean aphid feeding, suggesting that the lack of response in resistant soybeans may be due to suppression by aphids^53^. In combination, these studies support the hypothesis that induction of JA-related transcripts upon aphid feeding are not conditional of tolerance. Instead, the constitutive expression of JA-transcripts such as lipoxygenases is a more important component of plant tolerance to aphids.

While constitutive levels of ABA, JA, and JA-Ile were significantly higher in tolerant soybean, no differences in SA and OPDA were observed between genotypes pre- and post-aphid infestation. The slight down-regulation of constitutive SA in KS4202 may be due to the high expression of ABA and JA through known mechanisms of JA/SA antagonism^61,62^ and ABA suppression of SA-mediated responses in soybean^63^. In addition, the expression of *PATHOGENESIS-RELATED1* (*PR1*), an SA-responsive transcript, was down-regulated in KS4202 relative to K03-4686^47^, which is consistent with our findings on *PR1* constitutive expression. The early induction of SA after 6 hpi in both tolerant and susceptible genotypes suggest its accumulation is a generalized plant response to aphids. Selig *et al*. reported similar findings with the expression of genes downstream of SA biosynthesis (*PR1* and *PR2*) being cyclical in susceptible soybean including induction occurring after 6 h of soybean aphid feeding followed by a sudden decline^54^. Interestingly, soybean aphid feeding on soybean induced the production of methyl SA (MeSA) that attracts predatory beetles to the host plants, thereby limiting aphid proliferation^64^. However, our results confirm that, although MeSA contributes to indirect defenses in soybean^64^, SA is not a major contributor to soybean aphid tolerance in soybean.

In addition to JA and SA, ET is also a common hormonal response involved in plant-insect interactions. Marimuthu and Smith (2012) have proposed that constitutive expression of ET-responsive transcripts, in addition to the role of JA, is also important for aphid tolerance in barley^60^. Further, resistance to green peach aphids (*Myzus persicae*) in Arabidopsis also relies on the constitutive expression of JA or ET^65^. JA and ET have a synergistic relationship, and inhibition of ET biosynthesis can lead to a reduced accumulation of JA^66,67^. Although not quantified in this study, future research on the constitutive levels of ET and expression of ET-related transcripts in KS4202 will further contribute to a working model of tolerance to the soybean aphid.

Another possibility worth exploring is the prospect of WRKY transcription factors mediating pathway signaling in aphid tolerant soybean. Approximately 174 WRKY transcription factors have been identified to date in soybean, in which numerous subfamilies exhibit increased constitutive expression throughout different tissues^68^. WRKYs can interact as both negative and positive regulators of SA^69–71^. In soybean, several of these transcription factors provide increased resistance to the soybean cyst nematode (*Heterodera glycines*) and suggest SA-induced WRKYs may play a role in abiotic and biotic stress responses^68^. Many WRKY proteins, including *AtWRKY53*, act upstream of *NPR1* (*Non-expressor of PR genes 1*) and positively regulate its transcription^69,72^. NPR1 proteins are required for the activation of SA-responsive *PR* genes^71^ that encode small proteins that have antimicrobial or antifungal properties^68,73^. Overexpression of *WRKY53* induced PR proteins in wheat and reduced symptoms of pathogen infection in rice (*Oryza sativa*)^74^. The *PR2* (β-1,3-glucanase) and *PR3* (chitinase) transcripts and peroxidases have been associated with wheat antibiotic resistance to Russian wheat aphid^75^. Our data show higher constitutive expression of *PR1* in susceptible soybean, with only slight induction after 7 dpi. In tolerant soybean, however, changes in expression of *PR1* in response to aphid feeding are dependent upon the length of aphid infestation with rapid suppression at 6 hpi and 6x fold change induction at 7 dpi. Interestingly, changes in transcript expression of *PR1* mirrored aphid-induced changes in *PRX52* in tolerant soybean, suggesting that *PR1* and *PRX52* may be regulated by a common factor. Additionally, the soybean WRKY transcription factor *WRKY60* has been reported to be up-regulated in KS4202 after late aphid infestation, suggesting WRKY transcription factors may be responsible for mediating pathways involved in soybean aphid tolerance exhibited by KS4202^45,76^.

In *Arabidopsis thaliana, AtPAD4* encodes a nucleocytoplasmic protein required for defense against both pathogens and the green peach aphid^77,78^. *AtPAD4*-mediated resistance to the green peach aphid is independent of both *ENHANCED DISEASE SUSCEPTIBILITY1* (*EDS1*) and SA accumulation, which are required for pathogen resistance^79^. In soybeans, *GmPAD4* is also induced by soybean aphids, and may contribute to antibiosis in *Rag1* in the cultivar Dowling^51^. In our studies, aphid induction of *GmPAD4* was inconsistent where as its constitutive expression was lower in tolerant soybean. Taken together, these indicate that *GmPAD4* is not a key player in providing soybean tolerance to aphids, but instead may act as a key component in *Rag*-mediated antibiosis.

In some cases, the plant resistance to aphids is a quantitatively inherited trait^22^. Due to the complex nature of this category of resistance, it is highly likely that the tolerance may be controlled by quantitative trait loci (QTL) in plants. However, lack of genetically-closely related aphid tolerant lines pose a serious bottleneck in understanding the genetic basis of tolerance to aphids. A possible strategy to overcome this problem is to develop recombinant inbred lines (RILs), which can be potentially used as a permanent resource for mapping the tolerance traits^80^. Further, one of the biggest challenges in screening tolerant plants is to design a feasible method for evaluating tolerance phenotype. With the recent advancements in the area of high-throughput phenotyping^15,81^, we can now expedite screening for plants that are tolerant to aphids.

**Figure 5:**
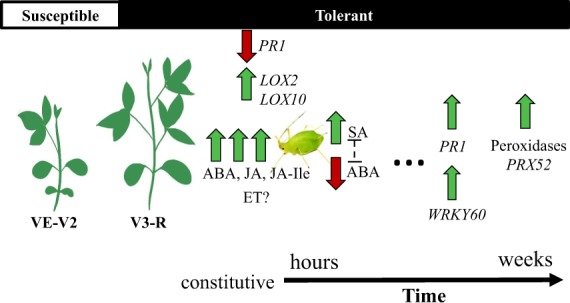
A working model of mechanisms involved in KS4202’s tolerance to the soybean aphid. Expression of tolerance in KS4202 is well defined during the V3 stage (fully developed leaf at third node^83^). High basal levels of ABA, JA, JA-Ile and defense-related lipoxygenases (*LOX*) contribute to susceptibility to aphid colonization and tolerance to aphid feeding-induced damage. The participation of ethylene (ET) warrants further research. Early aphid feeding induces SA and suppresses ABA. Over time with increasing aphid pressure, peroxidases become induced for the detoxification of accumulating ROS. Concurrently to peroxidases, *PR1*, and a transcription factor (*WRKY60*), are also induced by aphid feeding. Kait Chapman prepared the soybean illustration and we thank Ellis Johnson for the aphid illustration.

Overall, this research shows that constitutive elevated levels of JA and ABA is an important factor in soybean tolerance to soybean aphids (Fig. 5). Differently than the usual rapid transcriptional response observed in *Rag* soybeans^30,31^, induced transcriptional responses in tolerant soybean were slow and only detected later (>7 dpi). Nevertheless, primed induction of *PRX52*, *WRKY60*, and *PR1* transcripts are likely contributing to tolerance. The deployment of plant tolerance is an underexplored and valuable strategy that can mitigate the injury caused by soybean aphids. Our work provides important insights into the genotype of aphid-tolerant soybeans, as well as how these plants respond to aphid feeding. The recent development of EILs for KS4202^14^ in combination with the understanding of the tolerance mechanism provides a foundation for incorporating these plants in the IPM for soybean aphids. Besides the benefits of higher EILs, tolerance could also be used in association with *Rag*-soybeans. This would provide a more stable approach to manage soybean aphid population below economic damaging levels, and perhaps avoid substantial yield losses in the event of virulent aphid populations due to the tolerance background.

## Materials and Methods

### Plant and insect source

Aphid-tolerant soybean KS4202 (an F4 plant selection from KS4694 x C1842) and two aphid-susceptible genotypes (K03-4686 and Wyandot) were chosen to gain a better understanding of the differences in tolerance and susceptibility to aphid feeding. The genotype K03-4686 was previously used as the reference susceptible source in aphid tolerance studies^17,44,45,47^ and Wyandot (an F4 selection from the three-parent cross (Northrup King S29-18 × PI 274.421) × Ohio FG1) was included as a second susceptible source^82^.

All plants were grown in a growth chamber at 23 ± 2 °C under 15 L: 9D (light:dark) night and 75% humidity. Seeds were pre-germinated on wet paper towels enclosed in plastic bags and maintained at room temperature (24 ± 2 °C) for approximately 3 days. Seedlings were then planted in cone-tainers containing growing media and grown until V3 stage (fully developed leaf at third node)^83^, when the studies were initiated.

A soybean aphid colony was reared on KS4202 soybean in a growth chamber maintained at 24 ± 2 °C under a 16: 8 h (light: dark) photoperiod. Aphids were progeny of a Nebraska isolate, collected in fields at the University of Nebraska Haskell Agricultural Laboratory, Concord, NE (42°23′3″N, 96°29′21″W) in field season of 2011. As a colony maintenance procedure, soybean aphids were tested for virulence on soybeans expressing *Rag1* and *Rag2* traits; our tests indicated that the insects were susceptible to both of these resistance traits (i.e. biotype 1). Voucher specimens of soybean aphids were deposited in the Systematics Research Collections of the University of Nebraska State Museum (UNSM).

### Aphid bioassay and tissue collection

As no previous studies have shown early plant responses to soybean aphids in the aphid-tolerant genotype KS4202, a study was conducted to evaluate changes in transcript expression, hormone quantification, and peroxidase activity after 6 and 24 hours (early response study). In the early response study, V3 soybean plants were organized in a completely randomized factorial design including three soybean genotypes (KS4202, K03-4686, and Wyandot), two aphid infestation levels (0 [control] or 10 aphids per plant), and three evaluation time points (0, 6, and 24 hpi). Ten adult, apterous female aphids were placed on each newly expanded trifoliate. Infested trifoliates were then caged to prevent aphid escape. Cages were constructed with plastic petri dishes (8.9 × 2.5 cm) containing two mesh windows (7 cm diameter) and a small hole on the side to fit the petiole. A metal clip was placed on each side of the cage to secure the petri dishes. To provide cage support and prevent leaves from bending, a bamboo stick was cut to the appropriate height and placed in the potting soil. Each newly expanded trifoliate in uninfested (control) plants were also caged.

A second study, the late response study, was conducted to compare the overall changes in expression of defense related transcripts between tolerant and susceptible soybeans as a late response to soybean aphid feeding. Plants were completely randomized with a factorial treatment design that included two soybean genotypes (KS4202 and K03-4686), two soybean aphid infestation levels (0 [control] and 15 aphids per plant) and four plant evaluation/harvesting times (0, 1, 3, and 7 dpi). Plant trifoliates were then caged as previously described.

At each evaluation/harvesting time point, aphid number was recorded and removed from the plant with a soft paintbrush. Trifoliates were then excised and flash frozen in liquid nitrogen. Frozen tissue was ground with mortar and pestle and then separated into aliquots for RNA isolation and protein extraction, and hormone assays. Samples were stored at −80 °C until use.

### Plant hormone quantification

For the plant hormone measurements, three replications from the early response study (0 [control], 6, and 24 hpi) plants were randomly chosen from each genotype. LC-MS assay and quantification of plant hormones was performed by the Proteomics and Metabolomics Facility at the Center for Biotechnology/University of Nebraska-Lincoln. Hormones were extracted from approximately 100 mg of ground leaf tissue using cold methanol: acetonitrile (50:50, v/v). D5-1AA, D5-tZ, D5-tZR, D6-ABA, D2-JA, and D4-SA were used as deuterium-labeled internal standards for IAA, IAA-Asp, cZ, tZ, tZR, ABA, JA, JA-Ile, OPDA, and SA to account for experimental variation. ZORBAX Eclipse Plus C19 column flowing was used for LC separation and interfaced with a Sciex QTRAP 6500+ mass spectrophotometer equipped with a TurbolonSpray electrospray ion source. For quantification, an external standard curve was prepared. IAA, IAA-Asp, cZ, tZ, and ZR were not detected.

### Changes in transcript expression

Total RNA was isolated from approximately 300 mg of ground leaf tissue per sample with TRIzol reagent and treated with RNase-Free DNase I (Qiagen) for 10 min and followed by RNeasy MinElute Cleanup Kit (Qiagen, Valencia, CA) for purification. Purity and concentration of total RNA was determined with a spectrophotometer (NanoDrop 1000). cDNA was synthesized with 2.5 μg of RNA using ThermoScript RT-PCR system (Life Technologies) according to manufacturer’s protocol. Quantitative reverse transcription (qRT-PCR) reactions were performed on a 7500 Fast Real-time PCR (Applied Biosystems) using SsoAdvanced SYBR Green (Bio-Rad Laboratories, California, USA) following manufacturer’s protocol. Primers were designed using Primer-BLAST (National Center for Biotechnology Information) and sequences are provided in Supplemental Table 1. Soybean cyclophilin (*CYP*) was used as an endogenous control to normalize data^84^. Mean fold change from five biological replicates was calculated using the 2^−ΔΔCT^ as previously described^85^.

### Enzyme kinetics

Protein was extracted from ~100 mg of ground tissue per sample with Minute Total Protein Extraction for Plant Tissues (Invent Biotechnologies, Eden Prairie, MN). A protease inhibitor cocktail for plant tissue (Sigma-Aldrich, Saint Louis, MO) was sequentially added to each sample. Crude protein extract was added to 95% acetone and incubated for 1 hour at −20 °C. Samples were then centrifuged at 13,000 rpm for 10 minutes at 4 °C and supernatant was discarded. The derived pellet was allowed to air dry at room temperature and dissolved in 120 μL of 25 mM sodium hydroxide and the resulting solution was diluted in water at a 1:11 ratio. Soluble protein was quantified with the BCA protein assay (Pierce, Rockford) with bovine serum albumin as a standard. Samples were incubated at 37 °C for 30 min before absorbance was measured at 562 nm.

Peroxidase activity was determined by a modified protocol from Hildebrand *et al*.^86^ and Pierson *et al*.^44^. Each well of a microplate (96 wells) was loaded with 5 μL of plant extract. The reaction was started by adding 2.5 μL of 30% H_2_O_2_, 75 μL of 18 mM guaiacol, 25 μL of 200 mM HEPES buffer (pH 6.0) and 71.3 μL of distilled water in the well containing the undiluted plant extract. Enzymatic activity for five biological replicates for each treatment combination was measured as the increase in absorbance after 2 minutes at 470 nm in a spectrophotometer (BioTek PowerWave). The specific activity of total peroxidase was calculated using the molar absorptivity of guaiacol at 470 nm (26.6 × 10^3^ M^−1^ cm^−1^).

### Statistical analysis

To determine the impact of aphid feeding on the selected transcripts (CT values), hormonal quantification, peroxidase activity, and aphid bioassay a generalized mixed model analysis (PROC GLIMMIX, Cary, NC) was performed. Means were separated using Fisher protected least significant difference (LSD) procedure when appropriate (*P < *0.05).

## Electronic supplementary material

Supplementary Information

## Data Availability

The datasets generated during and/or analysed during the current study are available from the corresponding author on reasonable request.
