# Supplementary material for: Abscisic and Jasmonic Acids Contribute to Soybean Tolerance to the Soybean Aphid (Aphis glycines Matsumura)
Source: Sci Rep. 2018 Oct 11;8:15148. doi: 10.1038/s41598-018-33477-w (PMC6181993; doi:10.1038/s41598-018-33477-w)
Supplement: Supplementary file 1 — Supplementary Information [file 41598_2018_33477_MOESM1_ESM.pdf]

Supplementary Information

Abscisic and Jasmonic Acids Contribute to Soybean Tolerance to the Soybean Aphid (Aphis glycines Matsumura)

Kaitlin M. Chapman[1], Lia Marchi-Werle[1], Thomas E. Hunt[1], Tiffany M. Heng-Moss[1] and Joe Louis[1,2,*].

[1]Department of Entomology and [2]Department of Biochemistry, University of Nebraska-Lincoln, Lincoln, NE 68583

*****Corresponding author: Joe Louis
Department of Entomology and Department of Biochemistry
University of Nebraska-Lincoln
Lincoln, NE 68583, USA

Phone: (402) 472-8098
Fax: (402) 472-4687
E-mail: joelouis@unl.edu

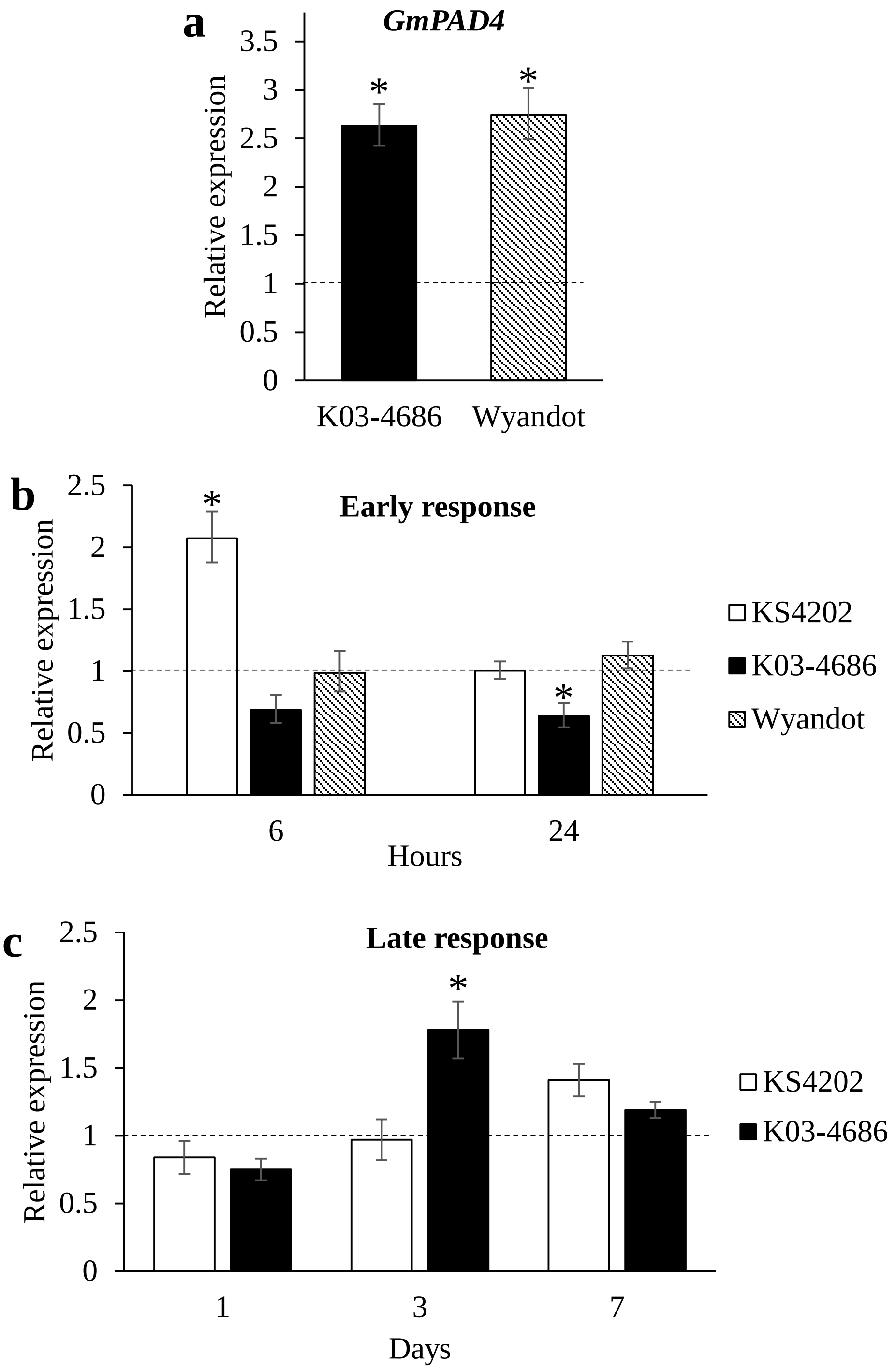

Supplemental Fig. 1. Constitutive and aphid-induced expression of *PAD4*. a) Constitutive *GmPAD4* expression in aphid-susceptible soybean lines relative to aphid-tolerant KS4202 with a baseline expression of 1. b) Early response study – Relative expression of *PAD4* in aphid-infested plants at 6 and 24 hpi. c) Late response study – Relative expression of *PAD4* in aphid-infested plants at 1, 3, and 7 dpi. Baseline expression in uninfested soybean is 1 for each genotype. Five biological replicates per treatment combination were used. (*) Indicates a significant difference between treatments and control. Error bars represent mean ± SE.

Supplemental Table 1. Primer sequences, accession numbers and locus for the genes targeted in this study.

Transcript	NCBI Accession Number	Locus	Primer Sequence
CYP	XM_014764426.1	GLYMA_12G024700	Fwd: ACGACGAAGACGAGTGG<br>Rev: CGACGACGACAGGCTTGG
LOX2	XM_003537901.3	GLYMA_11G130300	Fwd: ATGGAAATCAACGCGCTTGC<br>Rev: TGCAGGTCGAATTGCCATTG
LOX10	NM_001250409.2	GLYMA_08G189600	Fwd: TCTGCATCTCAAAATGTGATACCTC<br>Rev: CATCCATCCAGACAGATTCACTTG
OPR3	XM_003542310.3	GLYMA_13G109800	Fwd: GTGTATCAGCCTGGTGGG<br>Rev: GCACGAGGCTCTGGATAG
PR1	XM_003545723.3	GLYMA_15G062500	Fwd: AACTATGCTCCCCCTGGCAACTATATTG<br>Rev: TCTGAAGTGGTAGCTTCTACATCGAAACAA
NAC19	NM_001255827.1	GLYMA_13G030900	Fwd: TCGTTCACTATCTCTGCCGC<br>Rev: CCAACAGGTTTCGGTTTGCC
SCOF-1	NM_001248684.2	GLYMA_17G236200	Fwd: CCATCTTTTCCCTTTGACGA<br>Rev: GGTACTCTTCTTCAGAAGGATG
PRX52	NM_001254985.2	GLYMA_06G145300	Fwd: CCGCCATGATCAAGATGGGA<br>Rev: AACCCACCACGGAATCCAAA
WRKY60	NM_001251748.1	GLYMA_16G026400	Fwd: ATGGCAGCATGATGGATTCC<br>Rev: TTCTGTGCACGTTGACATGG
GmPAD4	NM_001255931.1	GLYMA_08G002100	Fwd: TGGAGGAAGCAAGGGACT<br>Rev: TCTTCAGACACCTCCTTATT
